# The impact of potentially inappropriate medication on the development of health care costs and its moderation by the number of prescribed substances. Results of a retrospective matched cohort study

**DOI:** 10.1371/journal.pone.0198004

**Published:** 2018-07-31

**Authors:** Dirk Heider, Herbert Matschinger, Andreas D. Meid, Renate Quinzler, Jürgen-Bernhard Adler, Christian Günster, Walter E. Haefeli, Hans-Helmut König

**Affiliations:** 1 Department of Health Economics and Health Services Research, Hamburg Center for Health Economics, University Medical Center Hamburg-Eppendorf, Hamburg, Germany; 2 Department of Clinical Pharmacology and Pharmacoepidemiology, University of Heidelberg, Heidelberg, Germany; 3 AOK research institute (WIdO), Berlin, Germany; University of Brescia, ITALY

## Abstract

**Background:**

In the growing population of the elderly, drug-related problems are considered an important health care safety issue. One aspect of this is the prescription of potentially inappropriate medication (PIM) which is considered to increase health care costs.

**Objective:**

Using data from the Health Economics of Potentially Inappropriate Medication (HEPIME) study, we aimed to analyze how the number of prescribed substances moderates the association of PIM use as defined by the German PRISCUS list and health care costs applying a longitudinal perspective.

**Methods:**

An initial number of 6,849,622 insurants aged 65+ of a large German health insurance company were included in a retrospective matched cohort study. Based on longitudinal claims data from the four separate quarters of a 12-month pre-period, 3,860,842 individuals with no exposure to PIM in 2011 were matched to 508,212 exposed individuals. Exposure effects of PIM use on health care costs and the number of prescribed substances were measured based on longitudinal claims data from the four separate quarters of the 12-month post-period.

**Results:**

After successful balancing for the development of numerous matching variables during the four quarters of the pre-period, exposed individuals consumed 2.1 additional prescribed substances and had higher total health care costs of 1,237 € when compared to non-exposed individuals in the 1st quarter of the post-period. Controlling for the number of prescribed substances, the difference in total health care costs between both study groups was 401 €. The average effect of one additionally prescribed substance (other than PIM) on total health care costs was increased by an amount of 137 € for those being exposed to a PIM. In quarters 2–4 of the post-period, the differences between both study groups tended to decrease sequentially.

**Conclusions:**

PIM use has an increasing effect on the development of health care costs. This cost-increasing effect of PIM use is moderated by the number of prescribed substances.

## Introduction

Particularly for older individuals, pharmacotherapy is a fundamental component of health care [1]. In Germany, 55% of all prescribed daily defined doses (DDD) are consumed by the subpopulation of those being aged ≥ 65 years which accounts for only 22% of the total population. Thereby, the average number of different drugs prescribed in a 3-month period in this age group is 4.6 [2]. The occurrence of age-related changes in pharmacokinetics and pharmacodynamics entails an increased risk of adverse drug reactions in the elderly [3]. This is even more important when older individuals are exposed to polypharmacy which, according to common definitions, is the use of five or more medications. Although the term polypharmacy was used to define overuse in the past [4], polypharmacy can be appropriate [5] and particularly its inappropriateness deserves special attention.

Concerning inappropriate prescriptions carrying more risks than benefits, the risk of adverse health outcomes is considered to be increased by the prescription of potentially inappropriate medications (PIMs) [6]. Adapted to the characteristics of the German drug market, the PRISCUS list was published in 2010 [7]. It is an systematically developed explicit PIM list that allows country-specific research on PIM-related topics and was largely inspired by the Beers-list in the U.S. or similar lists in other countries [8–11]. In the relevant age group of individuals ≥ 65 years, the prevalence of PIM use in Germany according to PRISCUS was estimated to be 25% [12].

PIM use in the elderly is associated with higher utilization of health services [6,13] which also reflects in higher health care costs [14,15]. Nevertheless, evidence on the topic is ambiguous [16]. This is probably caused by differences between relevant studies that involve methodological shortcomings like small and/or selective samples [17,18], retrospective cohort designs [19] and short follow-up periods [20]. Partly due to the lack of studies dealing with the longitudinal development of health care costs after PIM use, there is still doubt whether a causal relationship between PIM use and increased health care costs really exists. Meaningful and detailed analyses of the development of health care costs after PIM use have to involve the measurement of more than two observational occasions per individual. Because the risk of being exposed to PIM increases with the number of prescribed medications [21], these analyses should also carefully consider the moderating effect of the number of prescribed substances (in terms of Anatomical Therapeutic Chemical (ATC) codes [22] on the development of costs.

Based on data from a population of insurants aged ≥ 65 years from a large German statutory health insurance, and using an multiple-cell weighting [23] study design facilitated by a new statistical procedure called entropy balancing [24] that allows for the longitudinal balancing of large sample sizes, the present study addresses the following research questions:

1. How does the occurrence of a PIM use influence the development of health care costs longitudinally?
2. How does the occurrence of a PIM use influence the development of the number of prescribed substances (ATC codes) other than PIM longitudinally?
3. Does the development of the number of prescribed substances (ATC codes) moderate the association of PIM use and health care costs?

## Materials and methods

### Ethics

An ethical approval is not required as only routinely collected pseudonymized data are used. Privacy rights are guaranteed by a detailed data protection concept and do not require informed consent of the involved insurees.

### Study design and data

The retrospective matched cohort study design involves the application of entropy balancing [24] on routine claims data from the “Allgemeine Ortskrankenkassen (AOK)”, a large German statutory health insurance, covering approximately one third of the entire German population. Based on the total study population that consisted of 6,849,622 individuals aged **≥** 65 at the beginning of 2011, all individuals with a PIM prescription in 2011 according to the PRISCUS-list were assigned to the exposed group (EG). All other individuals were assigned to the non-exposed group (NEG). Afterwards, by excluding individuals of both study groups with a PIM-prescription during the 12-month pre-period, a “washout period” was established that ensures the inclusion of incident PIMs only. Then, individuals without prescription of any medication during that same period were excluded from both study groups. Finally, all individuals with any PIM prescription during the 12-month post-period in the NEG were excluded. Please see Heider et al. for a more detailed description of the study design and a summary of the inclusion/exclusion criteria [25]. In contrast to this earlier analysis [25], pre- and post-periods were each analysed on a quarterly basis in the present study. All analysed data were originally stored by fiscal quarters for the various health care sectors in the internal database of the health insurance funds. Thus, the observation of a total of 8 quarters per individual (4 in pre-period and 4 in post-period) allowed a precise analysis of the progression of health care costs and the number of prescribed substances before and after the occurrence of a PIM.

### Statistical analysis

#### PIM exposure and outcome variables

Except for tolterodine and nifedipine whose PIM definition depends on the drug formulation which was identified by their so-called German central pharmacy number (PZN) [26], PIM prescription was assessed by ATC codes [22] of all drugs on the PRISCUS list. Days in hospital, days in rehabilitation clinics, and the number of prescribed different drugs as determined by ATC codes were assessed for the evaluation of health service use. For medication consumption across all drugs, DDDs (including only those medications prescribed by outpatient physicians) were used as an indicator. For the evaluation of health care costs, costs of medication, inpatient treatment, outpatient physician services, medical supplies, and rehabilitation were separately measured from the perspective of the health insurance and later summed up to a variable that indicates total costs per individual.

#### Entropy balancing

A procedure aimed to estimate the unbiased effect of a PIM exposure by minimizing potential selection bias [27,28] was applied in two separate steps. The first step involved the balancing of the NEG with the EG using data from the 12-month pre-period using a statistical procedure called entropy balancing [24]. Entropy balancing has been shown to be superior to other approaches such as propensity score matching or pruning in terms of covariate balance [24,29]. A further special characteristic of the applied procedure is, that the calculation of the weighting vector was performed by incorporating the matching variables’ characteristics for the 4 separate quarters of the pre-period simultaneously. In the second step that is described more detailed in the section ‘regression analysis’, the weighting vector derived from pre-period was used to balance data of both study groups for all 4 quarters of the pre-period by means of regression techniques. This allowed for the balancing of a development (“growth curves”) for all matching variables during the whole 12-month pre-period between both study groups. Balancing was realized by using the software module ebalance [30] for STATA 14 [31]. A large number of covariates was used for balancing the data by means of the entropy balancing procedure. These 74 variables, including, among others, socio-demographic factors, participation in a disease management program (DMP) [32] and the Elixhauser Index [33, 34], are described in detail in S1 Text.

#### Regression analysis

Utilising the weighting vector gained from the entropy balancing procedure, in a second step linear mixture regression models with maximum likelihood estimators [35] were calculated to analyze the growth curves of the total health care costs and the numbers of prescribed ATC codes. Thereby, total health care costs and number of prescribed ATC codes were the dependent variables in separate, fully saturated statistical models. Independent variables included 7 dichotomous variables denoting the 8 quarterly periods of the study, one dichotomous variable for the study group (0 = NEG; 1 = EG) and the interaction terms between the group variable and all single quarterly period variables. Inspired by the idea of a so called four-group weighting by Stuart [23] for four independent groups, our approach would then be best called a multiple-cell weighting or more precisely a sixteen-cell weighting since two independent study groups with eight dependent time-points for each group are involved in our approach. We demonstrate the actual data structure underlying our analysis in a short example data set provided as S1 Table, since provision of the whole data set is not permitted due to legal restrictions.

**Fig 1:**
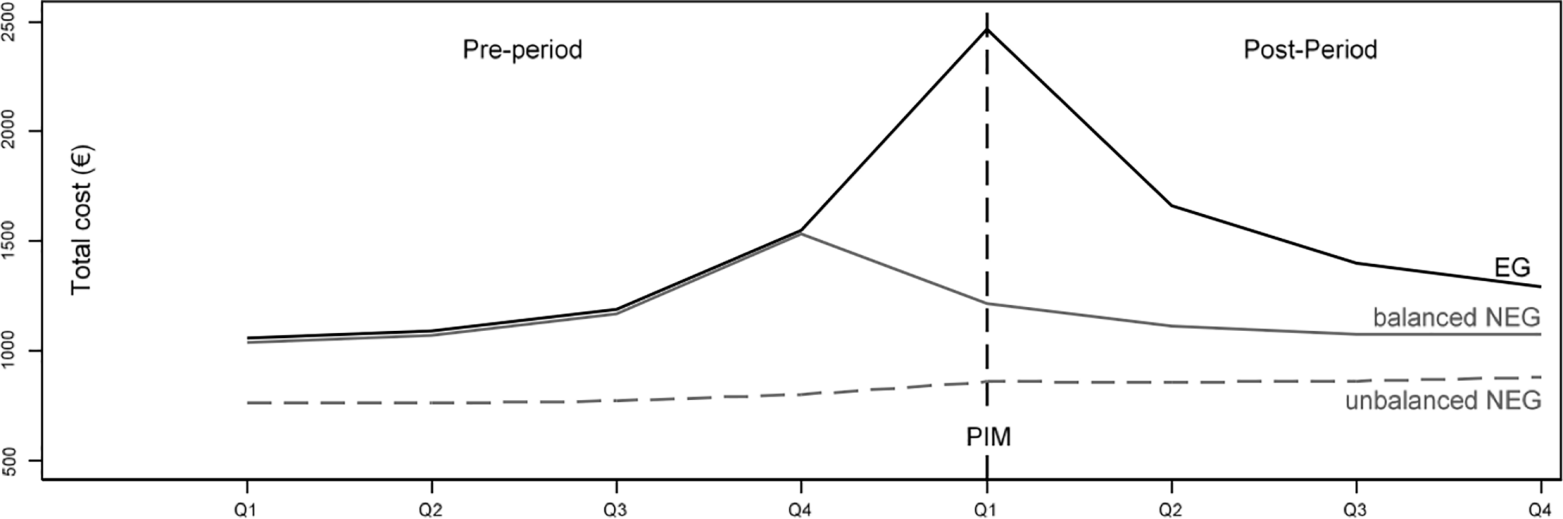
Growth curves for total health care costs (per quarter) in EG and balanced/unbalanced NEG for pre-and post-period, each spanning 4 quarters. Fig is based on fully saturated linear mixture regression model with maximum likelihood estimators, balancing of NEG is based on weights from entropy balancing.

**Fig 2:**
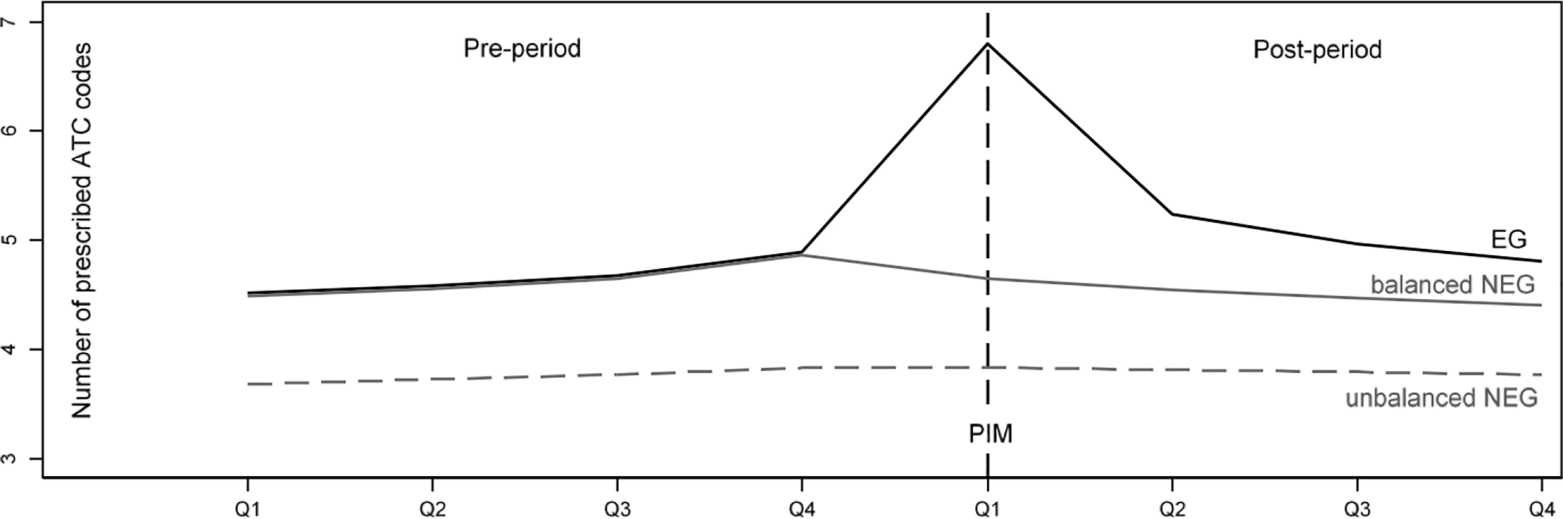
Growth curves for the number of prescribed ATC codes (per quarter) in EG and balanced/unbalanced NEG for pre-and post-period, each spanning 4 quarters. Fig is based on fully saturated linear mixture regression model with maximum likelihood estimators, balancing of NEG is based on weights from entropy balancing.

**Fig 3:**
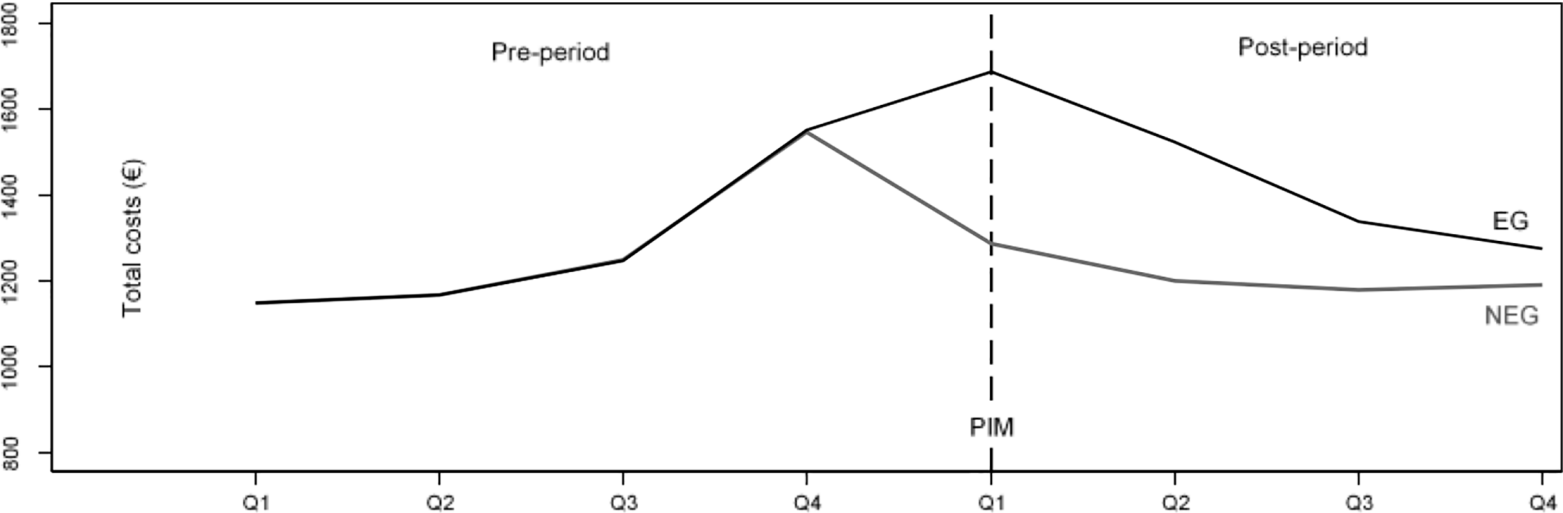
Predictive margins of growth curves for total health care costs in balanced EG and NEG, at mean of the number of prescribed ATC codes (mean 4.8), balanced by weights based on entropy balancing. Fig is based on fully saturated linear mixture regression model with maximum likelihood estimators with a quadratic term for the number of prescribed ATC codes.

To model the growth curve of the total health care costs controlled by the number of prescribed substances and suggested by goodness of model fit, an additional quadratic term for the number of prescribed ATC codes and its interactions with quarterly period and group variables was added to the list of independent variables. Furthermore, mean centering of this analysis by the number of prescribed ATC ensured that the influence of a PIM on health care costs is measured at a more realistic and reasonable point. Leaving the number of prescribed ATC codes uncentered would mean to calculate the influence of a PIM on health care costs for those individuals with a number of 0 prescribed ATC codes. An easier understanding is supported by graphical representations of the model results which include growth curves for the EG and balanced/unbalanced NEG (Figs 1–3).

For the calculation of moderating effects of the number of prescribed ATC codes and its square on costs of single health care sectors and aggregated total health care costs in the incident quarter of PIM use (first quarter of the post-period), equally structured statistical models were used (Table 1). The calculated moderating effects denote the different effects of the number of prescribed ATC codes on health care cost in both study groups, or put differently, the different effect of PIM (study group) at different values of the number of prescribed ATC codes and its square.

**Table 1:** Moderating effect of number of prescribed ATC codes on health care costs during first quarter of PIM use.

	NEG		EG		Difference			
Costs in €	Effect of ATC	(SE)	Effect of ATC	(SE)	Moderating effect of ATC	(SE)	p-value	R^2^
**Medication**	70.39	(0.36)	58.87	(0.46)	-11.52	(0.58)	0.000	0.164
**Outpatient physician services**	33.28	(0.17)	28.76	(0.21)	-4.47	(0.27)	0.000	0.107
**Hospital treatment**	79.54	(1.54)	240.04	(2.02)	160.50	(2.54)	0.000	0.007
**Rehabilitation**	5.40	(0.18)	15.03	(0.25)	9.63	(0.31)	0.000	0.007
**Medical supplies**	2.54	(0.04)	2.12	(0.05)	-0.42	(0.07)	0.000	0.014
**Total costs**	195.99	(1.66)	333.20	(2.18)	137.20	(2.74)	0.000	0.090

The calculation of moderating effects of the number of prescribed ATC codes in the incident quarter of PIM use is based on fully saturated linear mixture regression models with maximum likelihood estimators with a quadratic term for the number of prescribed ATC codes. Calculation of moderating effect of ATC is based on the interaction effect between the study group (EG vs. balanced NEG) and the number of prescribed ATC codes in the 1st quarter of the post-period. The statistical fit of the single models is shown in the last column in form of the Maddala-R^2^ which is based on the maximum-likelihood.

To achieve perfectly balanced growth curves for the pre-period in all statistical models the entropy balancing for the NEG weights were used as importance weights. Data management and data analysis were conducted using STATA 14.

## Results

### Sample characteristics

After applying the study’s inclusion/exclusion criteria, 2,374,555 individuals of the initial number of 6,849,622 individuals were excluded from further analysis. From the remaining individuals, 521,644 were assigned to the EG. The remaining 3,953,423 individuals with no PIM during follow-up were assigned to the NEG. Following the application of entropy balancing, an additional 106,013 individuals from the NEG were dropped from further analysis because they were lying outside the region of common support. This resulted in a total number of 4,369,054 individuals of whom 508,212 were assigned to the EG and 3,860,842 to the NEG. In the pre-period, 65% of individuals in the EG and 58% in the NEG were female before entropy balancing. The mean age was 75.9 years in the EG and 75.3 years in the NEG. The proportion of retired individuals was 94.6% in the EG and 94.0% in the NEG.

### Results of entropy balancing

Application of the entropy balancing procedure resulted in almost perfect balancing of EG and NEG in all four quarters of the 12-month pre-period. Shown by the numbers in S1 Table, means, standard deviations, and skewness of all matching variables in the NEG were adjusted to the corresponding values in the EG. This was achieved with a precision of two decimal places. The perfect balancing of the pre-period is visualized by boxplots for each of the first three statistical moments in S1 Fig, S2 Fig and S3 Fig where data points for the moments of the different matching variables in the NEG match to those in the EG after application of entropy balancing.

### Impact of incident PIM on development of health care costs

Fig 1 shows the development of total health care costs for both study arms over the 8 quarters. Visual inspection of the graph reveals successful balancing for the development of matching variables during the pre-period. There are no differences between the EG and the balanced NEG during all four quarters of the pre-period resulting in identical overlaying curves. Mean total costs steadily rise during the pre-period, starting at 1,059 € in the 1st quarter to 1,093 €, 1,193 €, and 1,555 € during the three subsequent quarters. With the occurrence of the incident PIM in the 1st quarter of the post-period the EG shows additional costs of 1,237 € (SE = 9.42) compared to the balanced NEG. The difference between both groups decreases during the subsequent quarters to amounts of 529 € (SE = 9.42), 300 € (SE = 9.42) and 196 € (SE = 9.42). Thereby, in the successive quarters of the post-period the mean number of prescribed PIMs in the EG was as follows: 1.06 (SD = 0.27), 0.30 (SD = 0.52), 0.25 (SD = 0.49), 0.23 (SD = 0.47).

### Impact of incident PIM on development of number of prescribed ATC codes

As displayed in Fig 2, the mean number of prescribed ATC codes rises slightly from 4.5 to 4.9 during the 4 quarters of the pre-period. Due to the balancing of the data, differences between the EG and the balanced NEG are virtually non-existent. After the occurrence of incident PIMs in the EG, 2.1 (SE = 0.006) additional ATC codes can be observed in the 1st quarter of the post-period, quickly decreasing to differences of 0.7 (SE = 0.006), 0.4 (SE = 0.006), and 0.4 ATC codes (SE = 0.006) in the subsequent quarters.

### Association between PIM and health care costs controlled by the number of prescribed substances

Fig 3 illustrates the development of total health care costs controlled for the number of prescribed ATC codes in both study groups. For this purpose, the number of prescribed ATC codes is centered at the mean of 4.8. Without significant differences between the EG and the balanced NEG, total costs consecutively rise from 1,149 € to 1,168 €, 1,249, and 1,550 € during the 4 quarters of the pre-period. In the 1st quarter of the post-period, total costs in the EG are 401 € (SE = 7.696) higher compared to the balanced NEG. This difference consecutively decreases over the following quarters of the post-period to 322 € (SE = 6.938), 160 € (SE = 6.932), and 84 € (SE = 6.944).

### Moderation of the association between PIM and health care costs by the number of prescribed substances

Because it follows from Figs 1 and 2 that differences in total health care costs and the number of ATC codes between the EG and the balanced NEG are largest in the 1st quarter of the post-period (i.e. the incident quarter of the PIM), the question of how the number of prescribed ATC codes moderates the association between an incident PIM and health care costs is addressed specifically in Table 1. The second column of Table 1 shows the increase in health care costs associated with the prescription of one additional ATC code during the 1st quarter of the post-period in the balanced NEG. The numbers in the fourth column of Table 1 represent the interaction effect between the study group (EG vs. balanced NEG) and the number of prescribed ATC codes in the 1st quarter of the post-period. According to Table 1, the prescription of one additional ATC code increases the total costs in the balanced NEG by an amount of 196 €, whereas for those being exposed to a PIM, the total costs increase by an amount of 333 € with every prescribed ATC code. Therefore, when compared with the balanced NEG, the effect of one additionally prescribed ATC code on total health care costs increases by an amount of 137 € for those being exposed to an incident PIM as indicated by the moderating effect of ATC in Table 1. This increase on total health care costs is a linear combination of a linear term with 133.3 € and 3.6 € for the quadratic term. The moderating effect on the difference in total health care costs is aggregated from the effects in the single health care sectors as follows: cost for hospital treatment: 161 €; cost for rehabilitation: 10 €; medication costs: -12 €; outpatient physician costs: -4.5 €; and costs for medical supplies: -0.4 €.

## Discussion

Our analysis clearly shows that the occurrence of a PIM influences the development of total health care costs longitudinally. While, during the perfectly balanced pre-period, the growth curves for health care costs in both study groups are practically identical, total health care costs in the exposed group increase more sharply with the occurrence of incident PIMs in the first quarter of the post-period pointing at a causal relationship. This difference gradually decreases with ongoing study duration induced by the now smaller number of individuals with a PIM prescription in the EG. The ascending slope of the costs during the pre-period even before the occurrence of a PIM can be most plausibly interpreted with the onset of health problems that precede the occurrence of a later prescribed PIM. In routine health insurance claims data, these health problems manifest in the utilization of health care services and increasing health care costs. Therefore, the ascending slope for health care costs during the pre-period in Fig 3 most likely indicates the occurrence of health problems that progress slowly, perhaps because they could not be treated adequately. So, the prescription of a PIM in the 1^st^ quarter of the post-period might be the last link in a chain of ongoing medical treatments and prescriptions that, especially in comparison to the balanced data of the NEG, strongly increases total health care costs in the EG. This is indicated by a clear spike in the EGs growth curve at this time point. This sudden increase of health care costs is likely to be the consequence of an acute worsening of the patients’ health status induced by the prescription of a PIM. It therefore may show the necessity for physicians to consider PIM lists when prescribing medications to patients with unstable and worsening conditions.

Evidence that the occurrence of a PIM influences the development of total health care costs longitudinally is even more substantiated by the observation that this becomes also visible when controlling for the number of prescribed ATC codes as illustrated in Fig 3. This also shows that the number of prescribed ATC codes moderates the association of PIM and total health care costs as well as costs in underlying sectors. Therefore, with every additionally prescribed ATC code, an average individual patient exposed to PIM exhibits additional quarterly total costs of 137 € (SE 2.74) when compared to an individual without PIM exposure. This finding indicates that beyond the cost-reducing potential of pure PIM lists, the number of prescribed substances per individual (in conjunction with PIM lists) has a high potential for reducing health care costs and thus is worth to be confirmed in a prospective clinical trial. In the 1^st^ quarter of the post-period, which is synonymous with the occurrence of the incident PIM, cost-increasing effects were found only for hospitalization and rehabilitation costs while, in all other cost sectors, the occurrence of incident PIMs has a lowering effect.

### Strengths and limitations

It is a major strength of the present study to have modeled a development of costs in the NEG and thereby answering the question what would have happened to the members of the EG if they had not been exposed to a treatment (PIM). The generalisation of this question is better known as the counterfactual in the Roy-Rubin model [27,28]. To our knowledge, the balancing of growth curves was not done in any preceding study. The applicability was largely owed to the availability of the newly developed statistical technique called entropy balancing which allowed very precise balancing of various patient baseline characteristics in a very large longitudinal sample.

One of the inherent limitations of German routine health insurance claims data that also limits our study’s findings is, that instead of the more precise information on actual PIM use (e.g. administration periods, adherence, and frequency of administration), only information on PIM prescriptions was available. Since only data from one health insurance were analysed, differences in socio-economic characteristics and morbidity between patients of various German health insurance funds may limit the representativeness of the data. Actually, insurants of the analysed health insurance are reported to have a lower socio-economic status and have a higher prevalence of chronical illnesses than insurants of other German health insurance funds [36]. Since we only analyzed routine health insurance (claims) data, there are no missing values in the sense of incomplete or refused questionnaires. However, there might be some misclassification bias or underreporting which represent well-known limitations for the use of health insurance data.

The assumption about causality between the prescription of a PIM and the various outcomes may have been somewhat limited by inaccuracies that result from the fact that there is a 90-day interval for an incident PIM prescription in the initial quarter of the study’s 12-month post-period. Therefore, the measured treatment effects may partially be attributable to PIM prescriptions that may have actually preceded the onset of the post-period. Nevertheless, in consideration of the very large sample size, the found treatment effects may have been only minimally affected by this inaccuracy.

It has further to be noted, that it would have been more realistic to compare individuals exposed to a PIM with individuals that are exposed to a list of safer therapeutic alternatives as attempted recently in a study by Endres et al. [13]. In the light of those listed limitations, the reported treatment effects are supposed to being slightly overestimated. A rather unclear influence on the magnitude of the observed treatment effect may have the so called unobserved heterogeneity. This means that despite the fact that we could draw from a comparatively large number of balancing variables it is possible that the study findings are partially attributable to differences in unobserved patient characteristics (e.g. health-related quality of life) or maybe prescribing practices of physician.

In subsequent studies, most of the mentioned limitations could be best dealt within observational epidemiologic studies, since the investigation of exposure to PIM in randomized controlled trials (RCT) is no realistic option due to ethical reasons. This would allow for the evaluation of the actual daily intake of PIMs rather than prescriptions based on quarters.

## Conclusions

By refining the previously used set of applied statistical methods [25], the present study provides further causal inference for the hypothesis that PIM use influences the development of health care costs and thus raises health care costs. This finding suggests a cost-reducing potential of PIM list implementations into medical routines. Furthermore, by showing that the cost-increasing effect of PIMs is also moderated by the number of prescribed substances, a second potentially cost-reducing factor could be identified. This moderating effect of the number of prescribed substances on the effect of PIMs should be taken into account when planning future prevention studies on the topic of PIM.

## Supporting information

S1 Fig Boxplot balancing of matching variables development in pre-period distribution of the means. (DOCX)

S2 Fig Balancing of matching variables development in pre-period distribution of variances. (DOCX)

S3 Fig Balancing of matching variables development in pre-period distributions of skewness. (DOCX)

S1 Table Data structure. (DOCX)

S2 Table Patients characteristics at baseline, pre-balancing (N = 4,369,054). (DOCX)

S3 Table Patients characteristics at baseline, post-balancing (N = 4,369,054). (DOCX)

S1 Text Matching variables. (DOCX)
