# Supplementary material for: The impact of potentially inappropriate medication on the development of health care costs and its moderation by the number of prescribed substances. Results of a retrospective matched cohort study
Source: PLoS One. 2018 Jul 31;13(7):e0198004. doi: 10.1371/journal.pone.0198004 (PMC6067698; doi:10.1371/journal.pone.0198004)
Supplement: S1 Fig — (DOCX) [file pone.0198004.s001.docx]

# S1 Figure: Boxplot balancing of matching variables development in pre-period distribution of the means

0

200

400

600

800

1,000

mean in treated units

mean in reweighted

control units

mean in raw

control units
