# Supplementary material for: The impact of potentially inappropriate medication on the development of health care costs and its moderation by the number of prescribed substances. Results of a retrospective matched cohort study
Source: PLoS One. 2018 Jul 31;13(7):e0198004. doi: 10.1371/journal.pone.0198004 (PMC6067698; doi:10.1371/journal.pone.0198004)
Supplement: S2 Fig — (DOCX) [file pone.0198004.s002.docx]

# S2 Figure: Balancing of matching variables development in pre-period distribution of variances

0

5.0e+06

1.0e+07

1.5e+07

variance in treated units

variance in reweighted

control units

variance in raw

control units
