# Supplementary material for: The impact of potentially inappropriate medication on the development of health care costs and its moderation by the number of prescribed substances. Results of a retrospective matched cohort study
Source: PLoS One. 2018 Jul 31;13(7):e0198004. doi: 10.1371/journal.pone.0198004 (PMC6067698; doi:10.1371/journal.pone.0198004)
Supplement: S3 Fig — (DOCX) [file pone.0198004.s003.docx]

# S3 Figure: Balancing of matching variables development in pre-period distributions of skewness

0

20

40

60

80

skewness in treated units

skewness in reweighted

control units

skewness in raw

control units
