# Supplementary material for: The impact of potentially inappropriate medication on the development of health care costs and its moderation by the number of prescribed substances. Results of a retrospective matched cohort study
Source: PLoS One. 2018 Jul 31;13(7):e0198004. doi: 10.1371/journal.pone.0198004 (PMC6067698; doi:10.1371/journal.pone.0198004)
Supplement: S1 Table — (DOCX) [file pone.0198004.s004.docx]

# S1 Table: Data structure

| ID | Study group | Study time | Study quarter | PIM occurrence | Total costs | Medication costs | Outpatient costs | Hospital costs | Rehabilitation costs | Medical supplies costs | Number of ATC | Entropy balancing vector |
| --- | --- | --- | --- | --- | --- | --- | --- | --- | --- | --- | --- | --- |
| A | NEG | Baseline | 1 | 0 | 70.66923 | 14.95 | 55.71923 | 0 | 0 | 0 | 1 | .0678827 |
| A | NEG | Baseline | 2 | 0 | 142.2153 | 27.24 | 114.9753 | 0 | 0 | 0 | 2 | .0678827 |
| A | NEG | Baseline | 3 | 0 | 70.76209 | 14.58 | 56.18209 | 0 | 0 | 0 | 1 | .0678827 |
| A | NEG | Baseline | 4 | 0 | 53.51058 | 14.58 | 38.93058 | 0 | 0 | 0 | 1 | .0678827 |
| A | NEG | Follow-up | 5 | 0 | 70.7238 | 14.58 | 56.1438 | 0 | 0 | 0 | 1 | .0678827 |
| A | NEG | Follow-up | 6 | 0 | 279.8026 | 28.13 | 251.6726 | 0 | 0 | 0 | 2 | .0678827 |
| A | NEG | Follow-up | 7 | 0 | 150.4251 | 10.88 | 139.5451 | 0 | 0 | 0 | 1 | .0678827 |
| A | NEG | Follow-up | 8 | 0 | 24.06702 | 0 | 24.06702 | 0 | 0 | 0 | 0 | .0678827 |
| B | EG | Baseline | 1 | 0 | 456.5396 | 195.35 | 159.7296 | 0 | 0 | 101.46 | 5 | 1 |
| B | EG | Baseline | 2 | 0 | 306.94 | 59.88 | 144.58 | 0 | 0 | 102.48 | 3 | 1 |
| B | EG | Baseline | 3 | 0 | 693.3215 | 102.33 | 590.9915 | 0 | 0 | 0 | 6 | 1 |
| B | EG | Baseline | 4 | 0 | 243.7138 | 91.56 | 152.1538 | 0 | 0 | 0 | 5 | 1 |
| B | EG | Follow-up | 5 | 1 | 9362.229 | 39.14 | 172.3079 | 7050.78 | 2100 | 0 | 4 | 1 |
| B | EG | Follow-up | 6 | 0 | 1207.085 | 71.2 | 331.2849 | 0 | 0 | 804.6 | 3 | 1 |
| B | EG | Follow-up | 7 | 1 | 284.7697 | 112.11 | 172.6597 | 0 | 0 | 0 | 3 | 1 |
| B | EG | Follow-up | 8 | 0 | 414.7328 | 178.95 | 235.7828 | 0 | 0 | 0 | 5 | 1 |

To illustrate the structure of the analysed data set, we provide the real data of two typical individuals (A and B), one of the NEG and one of the EG each. We chose the so called "long form" of data storage where each individual denoted by a unique "ID" variable is represented with 8 rows. Each of these rows represents a single "study quarter". The variable "study time" denotes if the study quarter belongs to the Baseline (0) or the Follow-up (1) period. This structure forms a two-level design (study quarters hierarchically nested within individuals) since the 8 "study quarters" indicate 8 dependent observations for each individual. This form of data is necessary to estimate the linear mixture regression. The variable "PIM-occurrence" denotes the occurrence of a PIM, as defined by the PRISCUS-List, with a "1" or "0" in case of PIM-occurrence or no PIM-occurrence during that quarter. Members of the NEG have "0" in all 8 rows. Members of the EG have "0" in all 4 rows of the baseline (washout) period and a "1" in the first row of the follow-up period indicating an incident PIM. They are allowed to have further values of "1" in the subsequent rows, indicating PIM-use after the incident occurrence of a PIM during the first quarter of the follow-up period. In the following columns are the various quarterly costs for the single individuals. Thereby, the total costs per row represent the sum of all sectoral costs (medication, outpatient, hospital, rehabilitation, medical supplies). In the next column is the number of (different) ATCs that an individual consumed during a quarter. The last column contains the vector calculated from the entropy balancing. This variable is constant across all 8 rows per individual. All members of the EG have a value of "1". This variable is used as a weighting variable in the regression analysis to balance both study groups. It is derived exclusively from the 4 quarters of the baseline period by using the list of matching variables, described in the methods section of the manuscript.
