# Supplementary material for: The impact of potentially inappropriate medication on the development of health care costs and its moderation by the number of prescribed substances. Results of a retrospective matched cohort study
Source: PLoS One. 2018 Jul 31;13(7):e0198004. doi: 10.1371/journal.pone.0198004 (PMC6067698; doi:10.1371/journal.pone.0198004)
Supplement: S3 Table — (DOCX) [file pone.0198004.s006.docx]

# S3 Table: Patients characteristics at baseline, post-balancing (N=4,369,054)

|  |  |  | **Exposed group (N = 508,212)** | | | **Non-exposed group (N = 3,860,842)** | | |
| --- | --- | --- | --- | --- | --- | --- | --- | --- |
|  |  | **Quarter** | **Mean** | **Variance** | **Skewness** | **Mean** | **Variance** | **Skewness** |
|  | **Sex** | **1** | 0.65 | 0.23 | -0.64 | 0.65 | 0.23 | -0.64 |
|  | **Age** | **1** | 75.85 | 52.09 | 0.48 | 75.85 | 52.09 | 0.48 |
| **Status of health care** | **Family member** | **1** | 0.00 | 0.00 | 25.60 | 0.00 | 0.00 | 25.60 |
|  | **Pensioner** | **1** | 0.95 | 0.05 | -3.94 | 0.95 | 0.05 | -3.94 |
| **Level of care** | **Level of care 1** | **1** | 0.85 | 0.13 | -1.94 | 0.85 | 0.13 | -1.94 |
|  | **Level of care 2** | **1** | 0.09 | 0.08 | 2.85 | 0.09 | 0.08 | 2.85 |
|  | **Level of care 3** | **1** | 0.05 | 0.05 | 4.18 | 0.05 | 0.05 | 4.18 |
| **Status of health care** | **Family member** | **2** | 0.00 | 0.00 | 26.76 | 0.00 | 0.00 | 26.76 |
|  | **Pensioner** | **2** | 0.95 | 0.05 | -3.94 | 0.95 | 0.05 | -3.94 |
| **Level of care** | **Level of care 1** | **2** | 0.84 | 0.14 | -1.84 | 0.84 | 0.14 | -1.84 |
|  | **Level of care 2** | **2** | 0.09 | 0.09 | 2.77 | 0.09 | 0.09 | 2.77 |
|  | **Level of care 3** | **2** | 0.05 | 0.05 | 3.99 | 0.05 | 0.05 | 3.99 |
| **Status of health care** | **Family member** | **3** | 0.00 | 0.00 | 29.94 | 0.00 | 0.00 | 29.94 |
|  | **Pensioner** | **3** | 0.95 | 0.05 | -3.94 | 0.95 | 0.05 | -3.94 |
| **Level of care** | **Level of care 1** | **3** | 0.83 | 0.14 | -1.73 | 0.83 | 0.14 | -1.73 |
|  | **Level of care 2** | **3** | 0.10 | 0.09 | 2.68 | 0.10 | 0.09 | 2.68 |
|  | **Level of care 3** | **3** | 0.06 | 0.05 | 3.80 | 0.06 | 0.05 | 3.80 |
| **Status of health care** | **Family member** | **4** | 0.00 | 0.00 | 35.83 | 0.00 | 0.00 | 35.83 |
|  | **Pensioner** | **4** | 0.93 | 0.07 | -3.27 | 0.93 | 0.07 | -3.27 |
| **Level of care** | **Level of care 1** | **4** | 0.81 | 0.15 | -1.58 | 0.81 | 0.15 | -1.58 |
|  | **Level of care 2** | **4** | 0.11 | 0.09 | 2.57 | 0.11 | 0.09 | 2.57 |
|  | **Level of care 3** | **4** | 0.07 | 0.06 | 3.52 | 0.07 | 0.06 | 3.52 |
| **Elixhauser (ICD-10 Quan et. Al.)** | **Congestive heart failure** | **All** | 0.22 | 0.17 | 1.33 | 0.22 | 0.17 | 1.33 |
|  | **Cardiac arrhythmias** | **All** | 0.26 | 0.19 | 1.08 | 0.26 | 0.19 | 1.08 |
|  | **Valvular disease** | **All** | 0.12 | 0.11 | 2.27 | 0.12 | 0.11 | 2.27 |
|  | **Pulmonary circulation disorders** | **All** | 0.04 | 0.03 | 4.99 | 0.04 | 0.03 | 4.99 |
|  | **Peripheral vascular disorders** | **All** | 0.19 | 0.15 | 1.61 | 0.19 | 0.15 | 1.61 |
|  | **Hypertension, uncomplicated** | **All** | 0.79 | 0.17 | -1.42 | 0.79 | 0.17 | -1.42 |
|  | **Hypertension, complicated** | **All** | 0.15 | 0.13 | 1.98 | 0.15 | 0.13 | 1.98 |
|  | **Paralysis** | **All** | 0.03 | 0.03 | 5.24 | 0.03 | 0.03 | 5.24 |
|  | **Other neurological disorders** | **All** | 0.07 | 0.07 | 3.24 | 0.07 | 0.07 | 3.24 |
|  | **Chronic pulmonary disease** | **All** | 0.26 | 0.19 | 1.07 | 0.26 | 0.19 | 1.07 |
|  | **Diabetes, uncomplicated** | **All** | 0.34 | 0.22 | 0.68 | 0.34 | 0.22 | 0.68 |
|  | **Diabetes, complicated** | **All** | 0.17 | 0.14 | 1.80 | 0.17 | 0.14 | 1.80 |
|  | **Hypothyroidism** | **All** | 0.11 | 0.10 | 2.42 | 0.11 | 0.10 | 2.42 |
|  | **Renal failure** | **All** | 0.13 | 0.11 | 2.22 | 0.13 | 0.11 | 2.22 |
|  | **Liver disease** | **All** | 0.13 | 0.12 | 2.16 | 0.13 | 0.12 | 2.16 |
|  | **Peptic ulcer disease excl. bleeding** | **All** | 0.03 | 0.03 | 5.43 | 0.03 | 0.03 | 5.43 |
|  | **AIDS/HIV** | **All** | 0.00 | 0.00 | 67.64 | 0.00 | 0.00 | 67.64 |
|  | **Lymphoma** | **All** | 0.01 | 0.01 | 9.31 | 0.01 | 0.01 | 9.31 |
|  | **Metastatic cancer** | **All** | 0.03 | 0.03 | 5.43 | 0.03 | 0.03 | 5.43 |
|  | **Solid tumor without metastasis** | **All** | 0.17 | 0.14 | 1.72 | 0.17 | 0.14 | 1.72 |
|  | **Rheumatoid arthritis/ CVD** | **All** | 0.10 | 0.09 | 2.66 | 0.10 | 0.09 | 2.66 |
|  | **Coagulopathy** | **All** | 0.04 | 0.04 | 4.93 | 0.04 | 0.04 | 4.93 |
|  | **Obesity** | **All** | 0.18 | 0.15 | 1.64 | 0.18 | 0.15 | 1.64 |
|  | **Weight loss** | **All** | 0.02 | 0.02 | 6.82 | 0.02 | 0.02 | 6.82 |
|  | **Fluid and electrolyte disorders** | **All** | 0.04 | 0.04 | 4.39 | 0.04 | 0.04 | 4.39 |
|  | **Blood loss anemia** | **All** | 0.01 | 0.01 | 13.40 | 0.01 | 0.01 | 13.40 |
|  | **Deficiency anemia** | **All** | 0.05 | 0.05 | 4.20 | 0.05 | 0.05 | 4.20 |
|  | **Alcohol abuse** | **All** | 0.02 | 0.02 | 6.35 | 0.02 | 0.02 | 6.35 |
|  | **Drug abuse** | **All** | 0.01 | 0.01 | 13.26 | 0.01 | 0.01 | 13.26 |
|  | **Psychoses** | **All** | 0.02 | 0.02 | 7.33 | 0.02 | 0.02 | 7.33 |
|  | **Depression** | **All** | 0.24 | 0.18 | 1.20 | 0.24 | 0.18 | 1.20 |
| **Disease management program** | **Coronary heart disease** | **1** | 0.11 | 0.10 | 2.55 | 0.11 | 0.10 | 2.55 |
|  | **Bronchial asthma** | **1** | 0.01 | 0.01 | 8.32 | 0.01 | 0.01 | 8.32 |
|  | **Diabetes mellitus type 2** | **1** | 0.20 | 0.16 | 1.54 | 0.20 | 0.16 | 1.54 |
|  | **Breast cancer** | **1** | 0.00 | 0.00 | 14.55 | 0.00 | 0.00 | 14.55 |
|  | **Diabetes mellitus type 1** | **1** | 0.00 | 0.00 | 30.80 | 0.00 | 0.00 | 30.80 |
|  | **COPD** | **1** | 0.03 | 0.03 | 5.25 | 0.03 | 0.03 | 5.25 |
|  | **Coronary heart disease** | **2** | 0.11 | 0.10 | 2.55 | 0.11 | 0.10 | 2.55 |
|  | **Bronchial asthma** | **2** | 0.01 | 0.01 | 8.29 | 0.01 | 0.01 | 8.29 |
|  | **Diabetes mellitus type 2** | **2** | 0.20 | 0.16 | 1.53 | 0.20 | 0.16 | 1.53 |
|  | **Breast cancer** | **2** | 0.00 | 0.00 | 14.61 | 0.00 | 0.00 | 14.61 |
|  | **Diabetes mellitus type 1** | **2** | 0.00 | 0.00 | 30.57 | 0.00 | 0.00 | 30.57 |
|  | **COPD** | **2** | 0.03 | 0.03 | 5.20 | 0.03 | 0.03 | 5.20 |
|  | **Coronary heart disease** | **3** | 0.11 | 0.10 | 2.55 | 0.11 | 0.10 | 2.55 |
|  | **Bronchial asthma** | **3** | 0.01 | 0.01 | 8.28 | 0.01 | 0.01 | 8.28 |
|  | **Diabetes mellitus type 2** | **3** | 0.19 | 0.16 | 1.54 | 0.19 | 0.16 | 1.54 |
|  | **Breast cancer** | **3** | 0.00 | 0.00 | 14.66 | 0.00 | 0.00 | 14.66 |
|  | **Diabetes mellitus type 1** | **3** | 0.00 | 0.00 | 30.69 | 0.00 | 0.00 | 30.69 |
|  | **COPD** | **3** | 0.03 | 0.03 | 5.19 | 0.03 | 0.03 | 5.19 |
|  | **Coronary heart disease** | **4** | 0.11 | 0.09 | 2.56 | 0.11 | 0.09 | 2.56 |
|  | **Bronchial asthma** | **4** | 0.01 | 0.01 | 8.31 | 0.01 | 0.01 | 8.31 |
|  | **Diabetes mellitus type 2** | **4** | 0.19 | 0.16 | 1.55 | 0.19 | 0.16 | 1.55 |
|  | **Breast cancer** | **4** | 0.00 | 0.00 | 14.73 | 0.00 | 0.00 | 14.73 |
|  | **Diabetes mellitus type 1** | **4** | 0.00 | 0.00 | 30.98 | 0.00 | 0.00 | 30.98 |
|  | **COPD** | **4** | 0.03 | 0.03 | 5.21 | 0.03 | 0.03 | 5.21 |
| **Federal State** | **Berlin** | **1** | 0.03 | 0.03 | 5.24 | 0.03 | 0.03 | 5.24 |
|  | **Brandenburg** | **2** | 0.04 | 0.04 | 4.62 | 0.04 | 0.04 | 4.62 |
|  | **Baden-Württemberg** | **3** | 0.15 | 0.13 | 2.00 | 0.15 | 0.13 | 2.00 |
|  | **Bavaria** | **4** | 0.16 | 0.14 | 1.82 | 0.16 | 0.14 | 1.82 |
|  | **Bremen** | **5** | 0.01 | 0.01 | 12.23 | 0.01 | 0.01 | 12.23 |
|  | **Hesse** | **6** | 0.06 | 0.06 | 3.70 | 0.06 | 0.06 | 3.70 |
|  | **Hamburg** | **7** | 0.01 | 0.01 | 10.02 | 0.01 | 0.01 | 10.02 |
|  | **Mecklenburg Western Pomerania** | **8** | 0.03 | 0.03 | 5.29 | 0.03 | 0.03 | 5.29 |
|  | **Lower Saxony** | **9** | 0.09 | 0.08 | 2.81 | 0.09 | 0.08 | 2.81 |
|  | **Northrine-Westphalia** | **10** | 0.15 | 0.13 | 1.96 | 0.15 | 0.13 | 1.96 |
|  | **Rhineland Palatinate** | **11** | 0.05 | 0.05 | 4.24 | 0.05 | 0.05 | 4.24 |
|  | **Schleswig Holstein** | **12** | 0.03 | 0.03 | 5.63 | 0.03 | 0.03 | 5.63 |
|  | **Saarland** | **13** | 0.01 | 0.01 | 9.33 | 0.01 | 0.01 | 9.33 |
|  | **Saxony** | **14** | 0.09 | 0.08 | 2.90 | 0.09 | 0.08 | 2.90 |
|  | **Saxony-Anhalt** | **15** | 0.05 | 0.04 | 4.37 | 0.05 | 0.04 | 4.37 |
| **Study quarter** | **Quarter** | **1** | 0.26 | 0.19 | 1.10 | 0.26 | 0.19 | 1.10 |
|  | **Quarter** | **2** | 0.24 | 0.18 | 1.20 | 0.24 | 0.18 | 1.20 |
|  | **Quarter** | **3** | 0.25 | 0.19 | 1.17 | 0.25 | 0.19 | 1.17 |
| **Adverse events** | **Hospital ^n^** | **1** | 0.01 | 0.01 | 9.95 | 0.01 | 0.01 | 9.95 |
|  | **Hospital ^c^** | **1** | 0.01 | 0.01 | 12.94 | 0.01 | 0.01 | 12.94 |
|  | **Outpatient ^n^** | **1** | 0.23 | 0.18 | 1.26 | 0.23 | 0.18 | 1.26 |
|  | **Outpatient ^c^** | **1** | 0.10 | 0.09 | 2.70 | 0.10 | 0.09 | 2.70 |
|  | **Rehabilitation ^n^** | **1** | 0.00 | 0.00 | 20.52 | 0.00 | 0.00 | 20.52 |
|  | **Rehabilitation ^c^** | **1** | 0.00 | 0.00 | 62.02 | 0.00 | 0.00 | 62.02 |
|  | **Hospital ^n^** | **2** | 0.01 | 0.01 | 9.62 | 0.01 | 0.01 | 9.62 |
|  | **Hospital ^c^** | **2** | 0.01 | 0.01 | 13.33 | 0.01 | 0.01 | 13.33 |
|  | **Outpatient ^n^** | **2** | 0.24 | 0.18 | 1.25 | 0.24 | 0.18 | 1.25 |
|  | **Outpatient ^c^** | **2** | 0.10 | 0.09 | 2.64 | 0.10 | 0.09 | 2.64 |
|  | **Rehabilitation ^n^** | **2** | 0.00 | 0.00 | 20.19 | 0.00 | 0.00 | 20.19 |
|  | **Rehabilitation ^c^** | **2** | 0.00 | 0.00 | 64.52 | 0.00 | 0.00 | 64.52 |
|  | **Hospital ^n^** | **3** | 0.01 | 0.01 | 9.03 | 0.01 | 0.01 | 9.03 |
|  | **Hospital ^c^** | **3** | 0.01 | 0.01 | 13.12 | 0.01 | 0.01 | 13.12 |
|  | **Outpatient ^n^** | **3** | 0.24 | 0.18 | 1.23 | 0.24 | 0.18 | 1.23 |
|  | **Outpatient ^c^** | **3** | 0.11 | 0.09 | 2.57 | 0.11 | 0.09 | 2.57 |
|  | **Rehabilitation ^n^** | **3** | 0.00 | 0.00 | 19.25 | 0.00 | 0.00 | 19.25 |
|  | **Rehabilitation ^c^** | **3** | 0.00 | 0.00 | 63.74 | 0.00 | 0.00 | 63.74 |
|  | **Hospital ^n^** | **4** | 0.02 | 0.02 | 6.97 | 0.02 | 0.02 | 6.97 |
|  | **Hospital ^c^** | **4** | 0.01 | 0.01 | 11.39 | 0.01 | 0.01 | 11.39 |
|  | **Outpatient ^n^** | **4** | 0.25 | 0.19 | 1.18 | 0.25 | 0.19 | 1.18 |
|  | **Outpatient ^c^** | **4** | 0.11 | 0.10 | 2.44 | 0.11 | 0.10 | 2.44 |
|  | **Rehabilitation ^n^** | **4** | 0.00 | 0.00 | 15.93 | 0.00 | 0.00 | 15.93 |
|  | **Rehabilitation ^c^** | **4** | 0.00 | 0.00 | 47.60 | 0.00 | 0.00 | 47.60 |
| **Health care service and costs** | **Number of different prescribed ATC classes** | **1** | 4.52 | 10.61 | 0.95 | 4.52 | 10.61 | 0.95 |
|  |  | **2** | 4.59 | 10.79 | 0.94 | 4.59 | 10.79 | 0.94 |
|  |  | **3** | 4.69 | 11.08 | 0.94 | 4.69 | 11.08 | 0.94 |
|  |  | **4** | 4.90 | 11.84 | 0.94 | 4.90 | 11.84 | 0.94 |
|  | **Medication costs** | **1** | 273.90 | 759247.00 | 26.45 | 273.90 | 759247.00 | 24.13 |
|  |  | **2** | 278.40 | 732034.00 | 17.57 | 278.40 | 732034.00 | 22.90 |
|  |  | **3** | 289.30 | 829726.00 | 19.99 | 289.30 | 829726.00 | 21.70 |
|  |  | **4** | 311.30 | 934540.00 | 13.53 | 311.30 | 934540.00 | 13.26 |
|  | **Number of prescribed DDD** | **1** | 390.80 | 128114.00 | 2.12 | 390.80 | 128114.00 | 2.12 |
|  |  | **2** | 397.00 | 129560.00 | 1.98 | 397.00 | 129560.00 | 1.98 |
|  |  | **3** | 404.70 | 132385.00 | 2.04 | 404.70 | 132385.00 | 2.04 |
|  |  | **4** | 415.20 | 135564.00 | 1.82 | 415.20 | 135564.00 | 1.82 |
|  | **Hospital treatment costs** | **1** | 497.10 | 5.80e+06 | 19.41 | 497.10 | 5.80e+06 | 19.41 |
|  |  | **2** | 523.00 | 6.74e+06 | 20.77 | 523.00 | 6.74e+06 | 20.77 |
|  |  | **3** | 603.20 | 8.89e+06 | 21.34 | 603.20 | 8.89e+06 | 21.34 |
|  |  | **4** | 914.30 | 1.57e+07 | 14.98 | 914.30 | 1.57e+07 | 14.98 |
|  | **Days in hospital** | **1** | 1.20 | 27.87 | 8.48 | 1.20 | 27.87 | 8.48 |
|  |  | **2** | 1.25 | 30.68 | 9.96 | 1.25 | 30.68 | 9.96 |
|  |  | **3** | 1.42 | 35.24 | 8.33 | 1.42 | 35.24 | 8.33 |
|  |  | **4** | 2.14 | 58.71 | 6.75 | 2.14 | 58.71 | 6.75 |
|  | **Outpatient physician services costs** | **1** | 228.70 | 305722.00 | 12.14 | 228.70 | 305722.00 | 12.14 |
|  |  | **2** | 231.60 | 318763.00 | 11.89 | 231.60 | 318763.00 | 11.89 |
|  |  | **3** | 237.90 | 333600.00 | 11.49 | 237.90 | 333600.00 | 11.49 |
|  |  | **4** | 254.50 | 366012.00 | 10.83 | 254.50 | 366012.00 | 10.83 |
|  | **Rehabilitation costs** | **1** | 28.66 | 118387.00 | 23.36 | 28.66 | 118387.00 | 23.36 |
|  |  | **2** | 28.25 | 124757.00 | 25.79 | 28.25 | 124757.00 | 25.79 |
|  |  | **3** | 30.59 | 136117.00 | 21.87 | 30.59 | 136117.00 | 21.87 |
|  |  | **4** | 41.59 | 206043.00 | 22.13 | 41.59 | 206043.00 | 22.13 |
|  | **Days in rehabilitation** | **1** | 0.23 | 5.72 | 13.74 | 0.23 | 5.72 | 13.74 |
|  |  | **2** | 0.22 | 5.48 | 13.59 | 0.22 | 5.48 | 13.59 |
|  |  | **3** | 0.23 | 5.88 | 12.84 | 0.23 | 5.88 | 12.84 |
|  |  | **4** | 0.30 | 7.73 | 11.56 | 0.30 | 7.73 | 11.56 |
|  | **Medical supplies costs** | **1** | 30.49 | 11893.00 | 8.32 | 30.49 | 11893.00 | 8.32 |
|  |  | **2** | 31.39 | 12338.00 | 7.75 | 31.39 | 12338.00 | 7.75 |
|  |  | **3** | 32.47 | 13215.00 | 7.86 | 32.47 | 13215.00 | 7.86 |
|  |  | **4** | 33.28 | 13678.00 | 8.26 | 33.28 | 13678.00 | 8.26 |

^n^ narrow definition of adverse events, ^c^ complementary definition of adverse events (the combination of ^n^ and ^c^ , forms a wide definition for adverse events)
