# Supplementary material for: The impact of potentially inappropriate medication on the development of health care costs and its moderation by the number of prescribed substances. Results of a retrospective matched cohort study
Source: PLoS One. 2018 Jul 31;13(7):e0198004. doi: 10.1371/journal.pone.0198004 (PMC6067698; doi:10.1371/journal.pone.0198004)
Supplement: S1 Text — (DOCX) [file pone.0198004.s007.docx]

### S1 Text: Matching variables

The covariates used for entropy balancing were sex, age, health insurance status (pensioner insurance, family insurance, or regular insurance), federal state of residence, and participation in a disease management program (DMP). In Germany, the term disease management is used for single disease programs which, based on findings from evidence-based medicine, focus on prevention and self-management of chronic conditions. DMPs are financed by insurance companies and include special arrangements for doctors and patients [32]. Other matching variables were the level of care (0 to 3, indicating the need for support in activities of daily living according to the German long-term care insurance), a set of 31 co-morbidities as defined by the Elixhauser Index [33,34], the occurrence of adverse events in a hospital, outpatient, or rehabilitation setting as well as health service use and health care costs within the 12-month pre-period. With respect to adverse drug reactions explicitly mentioned in the PRISCUS list, in the information from the German Summaries of Product Characteristics (SmPC), or in the ‘Meyler’s Side Effects of Drugs’ (19), non-fatal adverse events as previously used by Heider et al. [25] were assessed in the following four categories: 1. fractures (indicating falls), 2. adverse events affecting the central nervous system (CNS), 3. adverse cardiovascular events, and 4. bleeding. These adverse events were defined by the German modification of the International Classification of Diseases, tenth revision (ICD-10 GM) (20). Individuals with at least one match on any of these four categories were categorised as having an adverse event during follow-up. Dichotomous variables indicating the occurrence of at least one adverse event were defined separately for outpatient, hospital, and rehabilitation data.

Health care costs used for entropy balancing were classified into five separate cost sectors: medication, inpatient treatment, outpatient physician services, medical supplies, and treatment in rehabilitation clinics. Health service use measured in terms of the number of prescribed different ATC codes, DDD, days in hospital, and days in rehabilitation clinics was also used for entropy balancing. Considering the single categories of some of these variables, this adds up to a total number of 74 variables which were balanced using historical data from the 4 time points of the 12-month pre-period. To facilitate the presentation of such a large number of information, box plots as seen in S1 Figure were chosen for the presentation of the distributions of means for all 74 variables in the four quarters of the pre-period. Because entropy balancing allows also for the balancing of standard deviation and skewness the corresponding information is given in S2 Figure and S3 Figure.
